# Obesity-Related Asthma: Structure and Inflammation

**DOI:** 10.1007/s11882-026-01302-4

**Published:** 2026-10-14

**Authors:** Tony H. Tzeng, Pauline Kamau, Erick Forno, Kirsten M. Kloepfer

**Affiliations:** Pediatric Pulmonology, Allergy/Immunology, and Sleep Medicine, Department of Pediatrics, Indiana University School of Medicine and Riley Hospital for Children, Indianapolis, IN USA

**Keywords:** Obesity, Asthma, Obesity-related asthma, Airway inflammation, Dysanapsis

## Abstract

**Purpose of Review:**

Asthma and obesity are affecting children and teenagers at higher rates than previous generations. Epidemiological data link obesity during childhood with the subsequent development of asthma. In this review we describe developmental and inflammatory factors that contribute to asthma severity in children with obesity.

**Recent Findings:**

Asthma driven by obesity is likely due to both structural and inflammatory changes. Excess weight places mechanical stress on the airway thereby reducing lung volumes. In addition, obesity is linked to chronic inflammation. Adipose tissue releases pro-inflammatory cytokines (IL-6 and TNF-α) which drives the airway towards hyperresponsiveness. This “obesity-related asthma” phenotype does not respond well to corticosteroids making these patients difficult to treat. In children and teenagers, treatment options are limited to currently approved asthma medications; however, improvement in asthma control has been observed in adults utilizing metformin and GLP-1 agonists.

**Summary:**

Children with obesity are more likely to develop asthma. Their asthma symptoms tend to be more frequent and harder to control. With the rate of obesity on the rise, it is paramount to identify the mechanisms underlying systemic inflammation in those with elevated BMIs. This will allow us to find better treatment options for “obesity-related asthma.”

## Introduction

**Fig. 1:**
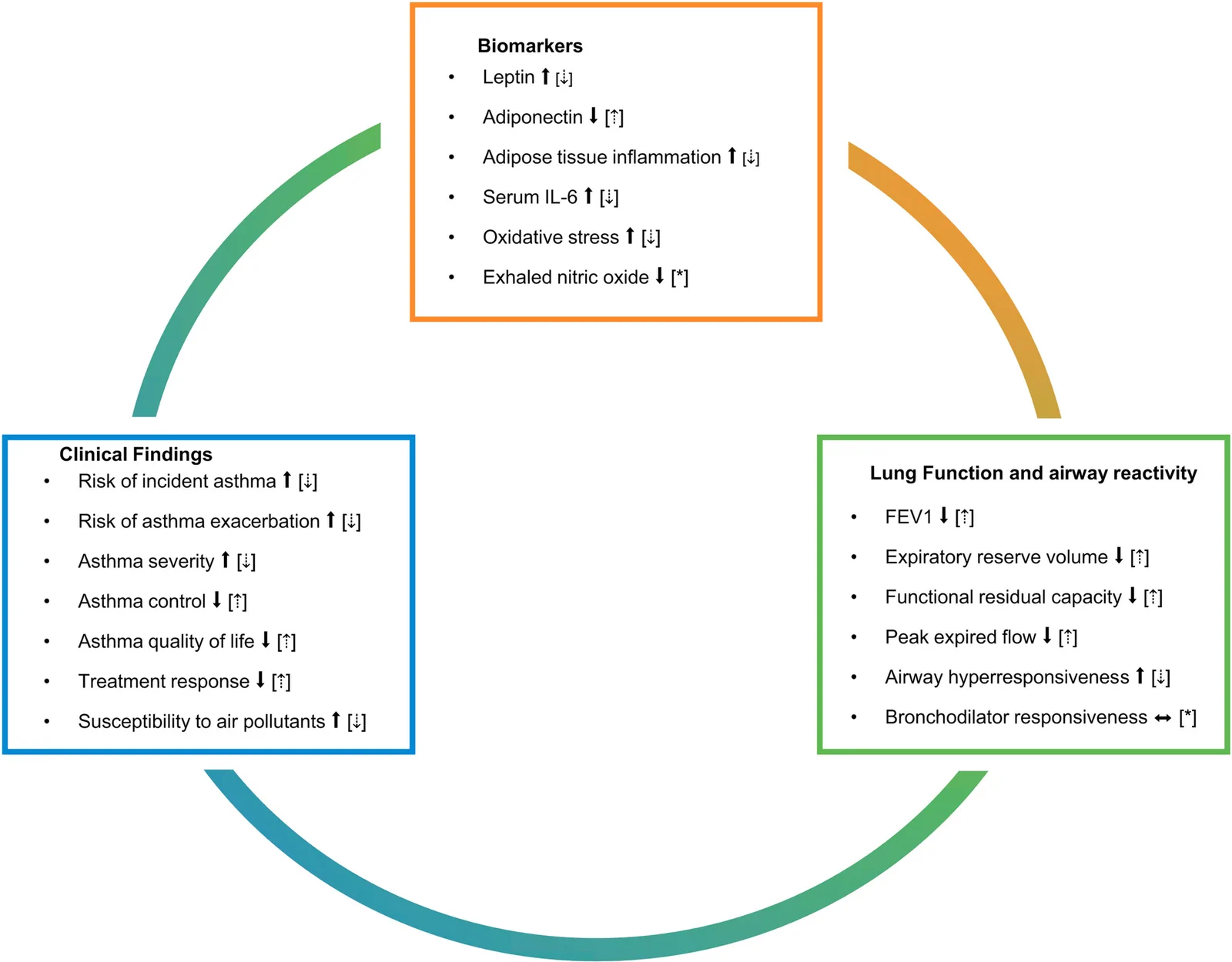
Effect of obesity on biomarkers and clinical outcomes of asthma. Proposed pathways that are altered in obesity and asthma. Solid arrows = effect of obesity. Dashed arrows inside brackets = changes seen after weight loss. Star = pathway has not been studied or no significant associations have been reported. Reproduced with permission from Peters U, Dixon AE, Forno E, J Allergy Clin Immunol 2018 (PMID 29627041) [4]

Obesity and asthma are both major public health problems in pediatrics, each affecting millions of children worldwide. In the U.S., asthma affects over 5 million children, while obesity affects almost 15 million youths ages 2–19 years [1, 2]. Many longitudinal epidemiological studies have associated childhood overweight/obesity or rapid weight gain with increased risk of subsequent incident asthma (later in childhood or in adulthood) [3–5]. There is evidence that even parental preconception BMI trajectories and maternal weight gain during pregnancy are associated with higher risk of asthma in their offspring [6, 7]. Moreover, in some children with asthma, obesity likely contributes to higher asthma severity and morbidity [8–11] –a phenotype of “obesity-related asthma” that may have significant management implications (Fig. 1) [12, 13]. Obesity-related asthma is a complex phenotype, and here we will focus on developmental and inflammatory factors that may contribute to asthma severity in these children.

## Growth Trajectories, Lung Function, and Asthma

### Trajectories of Obesity During Childhood

Early infancy and the preschool years are a period of significant growth, and children who experience rapid weight gain are at higher risk of developing obesity in later childhood and adolescence [14]. This coincides with a critical time window for asthma development: over 80% of cases of childhood asthma manifested symptoms before age 6 years, and rapid weight gain in the first three months of life has been associated with higher risk of subsequent asthma [15, 16]. A second window of increased body mass index (BMI) can be seen in middle childhood due to an increase in the number of adipocytes (hyperplasia). Children who experience an increase in adiposity between the ages of 4 and 7 years have a higher risk of later developing obesity [17, 18]; rapid weight gain at this age has also been linked with differences in lung function by adolescence [16]. A final period of significant risk for obesity happens during and after puberty: adolescents with obesity are likely to experience weight fluctuations and maintain an unhealthy BMI into their adult life [19]. This is also an important window for asthma, in which there can be worsening of asthma symptoms and control. Moreover, this coincides with the change in predominance from males (before puberty) to females (after puberty) [20]. While interventional evidence in pediatrics is scarce, preventing the development of obesity in these crucial periods could reduce the risk of asthma.

Longitudinal studies have described various weight trajectories in childhood. Rapid weight gain in the first 15 months of life can predict greater risk of a continued rise in BMI, which in turn is associated with greater risk of obesity by adolescence compared to children with stable BMI trajectories [21]. In a large study of the ECHO Cohorts including almost 10,000 children in the continental U.S. and Puerto Rico, two BMI trajectories were identified in the first 9 years of life: the “typical” trajectory was characterized by normative BMI decline after the first year of life up to 5–7 yrs of age, with a steady increase thereafter, while the “atypical” trajectory showed early and rapid BMI increases [22]. A similar trend among European infants followed into adolescence demonstrated a weight trajectory of “early high BMI” with eventual overweight status, compared to four other BMI trajectories that ultimately remained in the normal weight range [23]. How these BMI trajectories impact asthma severity is described in more detail in the following section.

### Association Between BMI, Lung Function Trajectories, and Asthma

**Fig. 2:**
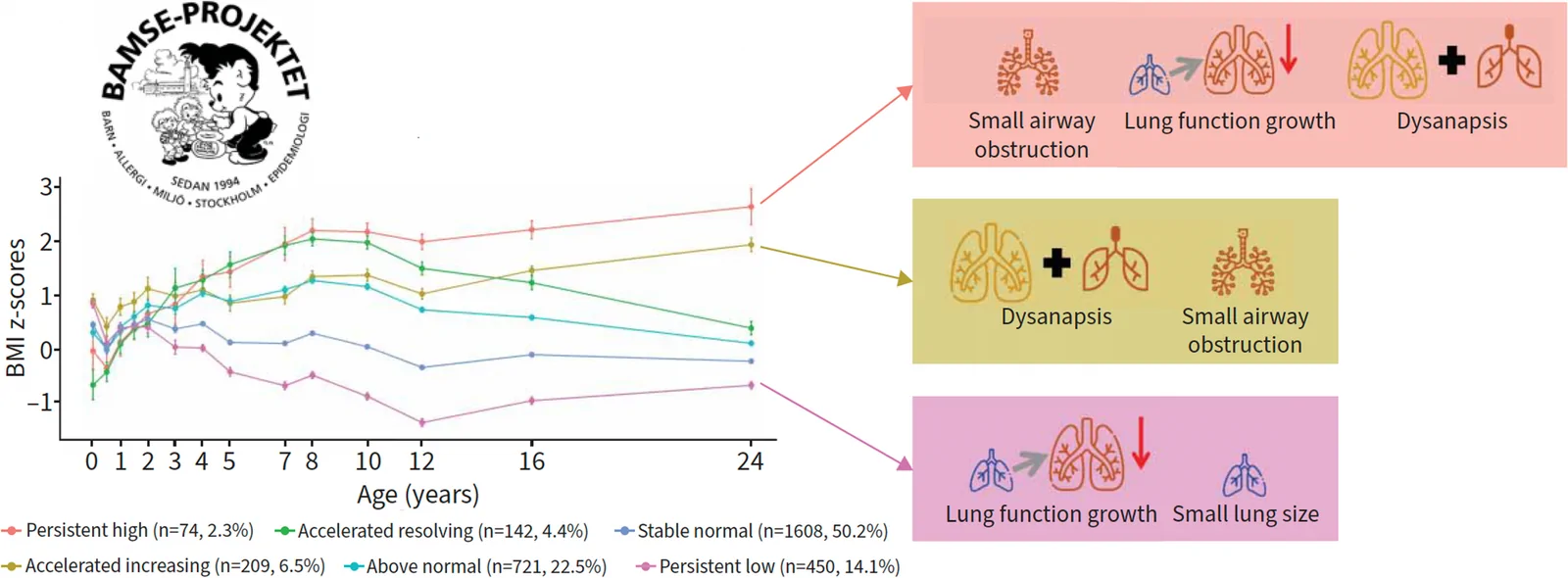
BMI trajectories from birth to early adulthood and lung function development. Six trajectories of BMI from birth to adulthood in the BAMSE cohort, and their impact on lung function development. Reproduced with permission from Wang G et al, Eur Respir J 2025 (PMID 39467611) [24]

Much like “growth charts” seen for height, weight, and BMI, lung function also follows clear growth trajectories from childhood through adulthood. BMI trajectories are associated with differences in lung function growth paths, and this may be more pronounced in youth with asthma. In a study of the BAMSE birth cohort in Sweden, investigators identified six different BMI trajectories form birth through age 24 years [24]. compared with youth who followed a normal BMI path, those in the “persistent high” and “increasing” BMI groups had signs of airflow obstruction, with low FEV1 or FEV1/FVC and an elevated lung clearance index (LCI) (Fig. 2) [24]. The LCI measures ventilation inhomogeneity, which is higher in severe asthma and correlates with lower FEV1 [25]. Interestingly, those who had “accelerated but resolving” BMI did not have altered lung function, suggesting that persistent obesity is more likely to impact lung function, and that interrupting abnormal BMI trajectories leading to obesity may reduce that impact. In a large analysis of airflow trajectories that included six European birth cohorts with BMI and spirometry data from childhood through early adulthood (ages 8 through 26 years), investigators found that individuals with asthma who maintained higher BMI exhibited lower likelihood of being in the lung function groups whose airflow obstruction improved over time [26]. Other studies have reported similar findings, and some have reported differential behaviors by sex and/or birthweight [27, 28].

Dysanapsis is an asymmetry in the growth of the airway caliber and the volume of the lungs. An analysis in six independent cohorts of children with and without asthma found that obesity was associated with spirometric evidence of airway dysanapsis [29]. Further, an analysis of longitudinal cohorts found that children with persistent obesity had ~ 4-fold higher prevalence of dysanapsis than their peers who always maintained a normal BMI; moreover, among youth who had obesity and asthma, spirometric evidence of dysanapsis was associated with more frequent severe disease exacerbations requiring ED visits and courses of systemic steroids [29]. Interestingly, while early-adulthood findings suggest that obesity is linked to airflow obstruction or dysanapsis, cohorts with follow-up through 40–50 years of age suggest that high BMI trajectories are instead associated with a restrictive deficit. This has also been reported in adolescents [30, 31]. It is unclear whether these differences are due to the degree and persistence of obesity (i.e., do youth with obesity and dysanapsis eventually develop a restrictive deficit in adulthood) or if these profiles affect different populations (i.e., dysanapsis if obesity developed early in life vs. restriction if it develops later on). In addition, some studies have shown that, even after adjusting for BMI or percent body fat, insulin resistance and the metabolic syndrome are associated with lower FEV1/FVC in adolescents, and that this may be more pronounced in youth with asthma; as a result, teenagers with asthma and metabolic syndrome had FEV1/FVC that was ~ 12–13% lower than their peers without either condition [32]. These and other similar findings suggest that optimizing a healthy weight among children with wheezing may help improve lung health across the lifespan –and that maintaining a healthy metabolic profile may be at least as important as a normal BMI range.

### Beyond Airway Mechanics: Transcriptomic and Metabolic Dysregulation Pathways

The effect of BMI on the airways goes well beyond airway mechanics. Some studies have reported associations between placental gene expression and subsequent high BMI and overweight/obesity in the offspring by 4 years of age [33], suggesting that BMI trajectories leading to obesity are not solely determined by post-natal factors like the child’s diet. In the BAMSE cohort, metabolite pathway analysis found that histidine-related metabolites in urine were significant effect modifiers of the association between BMI trajectory, FVC, and FEV1/FVC [24]. Moreover, high BMI from childhood to adulthood has been associated with systemic inflammation, as measured by C-reactive protein, a finding that was significant for White and Black males and females [34]. As we previously mentioned, metabolic dysregulation –independent of BMI and body composition– can be associated with significant deficits in lung function in youth with asthma [32]. A recent analysis of high vs. low BMI trajectories in childhood found that the association between BMI and increased asthma incidence was mediated by methylation changes at four genes, including two genes previously implicated in IL-6 pathways [35]. Children with high BMI also tend to exhibit lower levels of FeNO, a marker of eosinophilic airway inflammation that is usually elevated in atopic asthma [36]. Taken together, these findings suggest that different inflammatory pathways may mediate the effects of obesity on airway development, inflammation, and hyper-reactivity. These inflammatory and immune changes will be covered in the next section.

## Inflammatory and Immune Changes in Obesity and Asthma

### Immune and Inflammatory Pathways in Obesity Related Asthma

Obesity-related asthma results in a phenotype driven by immunological changes affecting airway and systemic inflammation that is less responsive to the standard asthma treatments. Eosinophilic asthma classically involves inflammation driven through T-helper (Th)-2 cellular pathways; however, obesity may alter immune and inflammatory responses resulting in a non- or low-Th2 asthma with characteristic poor response to steroids [37]. Increased adipose tissue in obesity may also create or exacerbate a pro-inflammatory state, contributing to increased airway and systemic inflammation in obesity-related asthma [38, 39]. This section reviews the immunologic and inflammatory pathway changes in obesity-related asthma, integrating the latest updates in the field.

Immune response changes in obesity-related asthma include systemic monocyte activation, modulated innate lymphoid cell activity, Th1 polarization, and neutrophilic inflammation [39, 40]. Recent studies, delving further into immune cellular differences in obesity-related asthma elucidated roles for specific CD4 + T cell subtypes and identified alterations in adipose tissue immune cell distribution [41, 42]. Distinct CD4 + T cell subtypes were characterized using single cell transcriptomics and noted to have differential gene expression and subsequent signaling pathway upregulation. This upregulation was associated with either Cell Division Cycle 42 (CDC42) upregulation, which underlies Th1 inflammation, or with Th1 polarization, while also associated with steroid resistance [41, 43]. Of note, CDC42 levels are correlated with higher pediatric asthma exacerbation risk and severity [44]. Additionally, there is a higher proportion of protein tyrosine phosphate receptor type C (PTPRC or CD45) high Th2 cells in obese-related asthma that are associated with increased neutrophil degranulation, interferon signaling, and apoptosis [42]. This study also noted differences in CD4 + T cell gene expression specifically in FKBP5, IL-6 signaling and rho-signaling, which were previously associated with more severe asthma and increased expression of IL-7R, IL-32, and PARP [42]. Indeed, increased IL-7R expression in T cells correlates with steroid insensitivity, with a mechanistic inhibitor study demonstrating that inhibition of the PI3K pathway (but not MAPK pathway) is associated with improving steroid sensitivity response [45]. Visceral adipose tissue in obese adolescents was enriched with leukocytes, especially macrophages, and though a trend for increased proportion of CD4 + Th1 cells was noted, no differences in CD4 + T cells or other subsets were found [46]. These findings contribute to our understanding of the immunologic pathways in obesity-related asthma by identifying and characterizing specific T cell subtypes and other changes in immune cells, which may contribute to the Th1 polarization and decreased steroid sensitivity as well as overall disease severity seen in this asthma endotype.

The systemic inflammation in obesity involves systemic monocyte activation, with contributions from other inflammatory mediators including adipokines like leptin and pro-inflammatory cytokines, which may work to worsen both airway and systemic inflammation in obesity-related asthma [39, 47]. Indeed, one study reported that patients with both comorbid asthma and obesity had elevated leptin levels as well as pro-inflammatory cytokines including IL-6, TNF-a, IL-10, and IL-13 along with significantly longer hospital stays compared to asthma alone [48]. Interestingly, another study noted increased inflammatory mediators including IL-5, IL-17 A, IL-33, TNF-α, and leptin in both obesity and asthma but did not find a specific synergistic or additive interaction that affected cytokine levels [49]. Increased systemic inflammatory markers and alterations in amino acid metabolites reflective of oxidative stress, including glutamine, ornithine, and arginine, were found in a longitudinal study, but the exact relationship of oxidative stress and systemic inflammation in obesity remain unclear in pediatric obesity-related asthma [50]. These studies add to our base of knowledge yet highlight the complex and not yet completely understood interplay between the two inflammatory conditions underlying pediatric obesity-related asthma.

Overall, these findings updated our understanding of the complex and interconnected immunologic response in obesity-related asthma which is distinct from T2-high asthma. The combination of adipose tissue-driven inflammation in obesity with altered inflammatory and immune signaling pathways and airway remodeling contributes to the challenging clinical presentation in pediatric obesity-driven asthma. These recent advances help refine our understanding of the underlying pathophysiology, allowing us to identify new pathways of interest as well as potential therapeutic targets, such as in modulating T cell signaling and restoring steroid responsiveness.

### Future Directions and Potential Therapeutics

Although there are currently no therapeutics approved specifically for obesity-related asthma, treatments addressing other co-morbid conditions may improve asthma control. Metabolic dysregulation, insulin resistance, and dyslipidemia are comorbid conditions that likely exacerbate obesity-related inflammation, potentially through mechanisms such as altered insulin receptor expression in airway smooth muscles, and subsequently may contribute to the severity of certain phenotypes of obesity-related asthma [39]. Indeed, in a nation-wide study, young adults with obesity had significantly greater odds of concurrent metabolic syndrome, though they did not have worsening odds of self-reported asthma attacks/ED visits [51]. Currently, there are drugs that improve glycemic function in adults, including glucagon-like peptide-1 receptor agonists (GLP-1 agonists), and metformin that may also improve asthma control in obesity-driven asthma. Notably many of them remain either unstudied or understudied in adolescent and pediatric populations. The following section reviews the current clinical data for these treatments, though the literature is primarily in adults.

GLP-1 agonists are a class of pharmaceuticals growing in popularity in the management of obesity and diabetes for adults, with significant benefits in weight loss. This class improves glycemic control by acting on both insulin and glucagon regulation but may also have anti-inflammatory or immunomodulatory effects in the lungs, with subsequent effects on airway hypersensitivity and airway inflammation [52, 53]. GLP-1 agonists may have anti-inflammatory roles within the lung through both T2 and non-T2 pathways [12, 52]. Preclinical studies suggest that obese murine models pretreated with GLP-1 agonists have decreased airway eosinophils and pro-inflammatory cytokines [12]. Other preclinical studies found decreased Il-1β and NLPR3 inflammasome as well as decreased neutrophilic inflammation on BAL following GLP-1 agonist treatment [12]. GLP-1 agonists may directly suppress inflammatory cells as well as downregulate chemokines responsible for mediating immune effectors of allergic airway response [52]. In vitro studies with GLP-1 agonists also propose a mechanistic role for lung inflammation suppression through a protein kinase A dependent suppression of the NF-kB signaling pathway with subsequent reduced macrophage activation [52]. Early clinical data hint at potential effects of GLP-1 agonists in obesity-related asthma in adults with decreased exacerbations of asthma as well as improvements in FEV1 and FVC [52]. A meta-analysis in adults with obesity and diabetes who were taking GLP-1 agonists revealed a trend for asthma risk reduction in those with asthma. In subgroup analysis there was a significant asthma risk reduction in patients on light molecular weight GLP-1 agonists (e.g. semaglutide) [54]. GLP-1 agonists remain a promising therapeutic for obesity-driven asthma, which may have multimodal effects in addition to loss. These include improved glycemic regulation and reduction of lung inflammation to improve asthma clinical outcomes and pulmonary function. However, currently, there are no published major clinical studies in adolescent or pediatric populations.

**Fig. 3:**
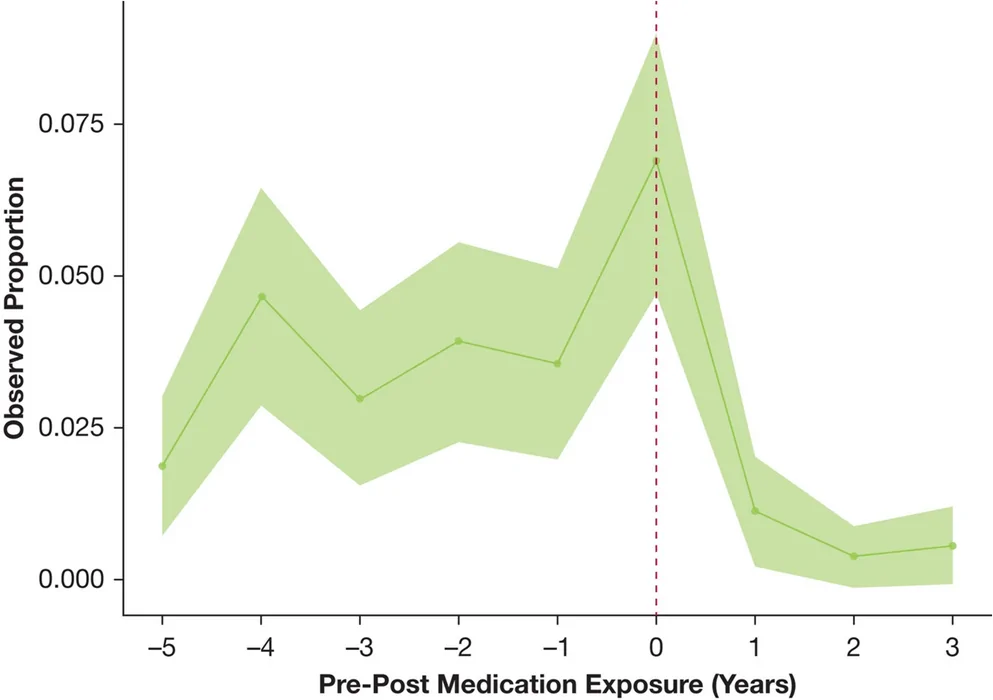
Severe exacerbations before and after initiation of metabolic-related medications in youth with asthma. The figure shows the observed proportions (and 95% confidence interval) of patients with an asthma-related hospitalization or ED visit before and after starting a metabolic medication (at time 0). Reproduced with permission from Aguree S et al, CHEST Pulm 2025 (PMID 40936793) [61]

Metformin, the first-line agent for insulin resistance in adults with diabetes, has been found in primarily retrospective studies to decrease ED visits and exacerbations in patients with asthma; and decreased asthma incidence [53]. Mechanistic studies in an obese asthmatic murine model suggests that metformin may modulate the AMPK/NLRP3 axis to decrease downstream inflammatory mediators including leptin and IL-1/IL-18 [55]. Supporting these mechanistic studies is a clinical study in adults that found metformin treatment significantly changed levels of oxidative stress and steroid sensitivity. These studies hint at a potential mechanistic effect on oxidative stress with subsequent improvement in steroid sensitivity [56]. Interestingly, in a large retrospective study in adults, metformin use was associated with lower rates of asthma exacerbations, without modifying effect from hemoglobin A1c or BMI changes, hinting at mechanisms beyond weight and glycemic control [57]. In support of these findings, a recent study noted comparable effects of SGLT-2 inhibitors compared to metformin in adults with a decrease in asthma exacerbations requiring systemic corticosteroids [58]. While data exists supporting metformin’s influence on asthma, little is known about the effect on the pediatric population. Two recent retrospective studies in the adolescent and pediatric population reported decreased asthma exacerbation rates after metformin prescription as well as significantly decreased rate of systemic corticosteroid courses [59, 60]. Another recent study showed that initiation of metabolic or anti-diabetic medication in youth with asthma may lead to significant reductions in acute asthma exacerbations (Fig. 3) [61]. Overall, metformin is well studied in the adult population, with some initial studies in the pediatric and adolescent age group that hint at potentially similar beneficial effects on asthma control.

## Conclusions and Future Directions

With rates of obesity on the rise, it is important to fully understand the influence that an elevated BMI has on systemic inflammation. Children with obesity are significantly more likely to develop asthma, and when they do, their symptoms tend to be more frequent and harder to control. This is likely due to both structural and inflammatory changes. Excess weight places mechanical stress on the airway which reduces lung volumes, thereby leading to breathing difficulty. In addition, obesity is linked to chronic inflammation. Adipose tissue releases pro-inflammatory cytokines (IL-6 and TNF-α) which drives the airway towards hyperresponsiveness. This “obesity-related asthma” phenotype does not respond well to corticosteroids making these patients difficult to treat.

Treatment options for “obesity-related asthma” are limited. As discussed in this review, patients with BMI’s that improve over time have improvement in their lung measurements, supporting weight loss as a mode of treatment. While weight loss can be an effective answer, it can be a difficult feat for patients of all ages. Newer studies support metformin as a possible option in the pediatric population as it has been shown to decrease asthma exacerbation frequency and oral corticosteroid utilization in adolescents. In adults, the newer GLP-1s and SGLT-2s show promising results in reducing asthma exacerbations and should be studied in adolescents and school-age children as a possible adjunct therapy while working to modify diet and exercise habits. Continued studies are needed to help us define biomarkers associated with steroid-resistant obesity-related asthma. This will allow us to target specific inflammatory markers with treatment and avoid permanent airway changes.

## Key References

- Liu C, Chow SM, Aris IM, Dabelea D, Neiderhiser JM, Leve LD, Blair C, Catellier DJ, Couzens L, Braun JM, Ferrara A, Aschner JL, Deoni SCL, Dunlop AL, Gern JE, Rivera-Spoljaric K, Hartert TV, Hershey GKK, Karagas MR, Kennedy EM, Karr CJ, Barrett ES, Zhao Q, Lester BM, Check JF, Helderman JB, O'Connor TG, Rasmussen JM, Stanford JB, Mihalopoulos NL, Wright RJ, Wright RO, Carroll KN, McEvoy CT, Breton CV, Trasande L, Weiss ST, Elliott AJ, Hockett CW, Ganiban JM, Environmental influences on Child Health O. Early-Life Factors and Body Mass Index Trajectories Among Children in the ECHO Cohort. JAMA Netw Open. 2025;8(5):e2511835. Epub 20250501. https://doi.org/10.1001/jamanetworkopen.2025.11835. PubMed PMID: 40402497; PMCID: PMC12100454.
  - ○ This study utilized data from over 10,000 children in the ECHO cohort to examine obesity trajectories in children. Authors were able to identify children on the path to obesity as early as age 3.5 years. These children had rapid growth between ages 3.5-9 years, with their BMI exceeding the 99% at age 9 years. This demonstrates that obesity starts early in life.
- Wang G, Hallberg J, Merid SK, Kumar A, Klevebro S, Habchi B, Chaleckis R, Wheelock CE, Hernandez-Pacheco N, Ekstrom S, Janson C, Kull I, Bergstrom A, Melen E. Body mass index trajectories from birth to early adulthood and lung function development. Eur Respir J. 2025;65(1). Epub 20250102. https://doi.org/10.1183/13993003.00298-2024. PubMed PMID: 39467611; PMCID: PMC11694551.
  - ○ This Swedish birth cohort followed over 3,000 infants through 24 years of age. Those with persistently elevated BMI during childhood and early adulthood had lower FEV1 and FVC z-scores. Those with an elevated BMI that decreased over time had PFT values that improved, suggesting that early interventions to lower BMI may contribute to improved lung health.
- Forno E, Han YY, Muzumdar RH, Celedon JC. Insulin resistance, metabolic syndrome, and lung function in US adolescents with and without asthma. J Allergy Clin Immunol. 2015;136(2):304-11 e8. Epub 2015/03/10. https://doi.org/10.1016/j.jaci.2015.01.010. PubMed PMID: 25748066; PMCID: PMC4530022.
  - ○ Insulin resistance and metabolic syndrome are associated with worsened lung function in overweight/obese adolescents. Asthma and metabolic syndrome synergistically decrease lung function, as do obesity and insulin resistance. These factors might contribute to the pathogenesis of asthma severity in obese patients and warrant further investigation.
- Tejwani V, Wang R, Villabona-Rueda A, Suresh K, Wu TD, Adcock IM, Kermani NZ, Zein J, Hansel NN, Yegnasubramanian S, McCormack MC, D’Alessio FR. Distinct single-cell transcriptional profile in CD4 + T-lymphocytes among obese children with asthma. American journal of physiology Lung cellular and molecular physiology. 2025;328(3). https://doi.org/10.1152/ajplung.00270.2024.
  - ○ This study identified unique contributors to asthma in children with obesity and found novel pathways. Increased expression of IL-7R, IL-32, PARP-1, FKBP5 gene, IL-6, and Rho transcriptional signaling were observed in obese individuals with asthma.
- Jiang W, Jiao R, Ma S, Dai J, Zhu H, Wu M, Che Y, Zhang L, Ding X. Serum inflammatory factors, vitamin D levels, and asthma severity in children with comorbid asthma and obesity/overweight: a comparative study - PubMed. Frontiers in pediatrics. 02/27/2025;13. https://doi.org/10.3389/fped.2025.1439841.
  - ○ Compared to those with asthma or obesity/overweight alone, children with asthma combined with obesity/overweight had significant differences in IL-6, IL-10, IL-13, 25-(OH) D3 levels, and leptin. Children with obesity/overweight and asthma had more severe clinical manifestations and longer hospital stays compared with those with asthma alone.

## Data Availability

No datasets were generated or analysed during the current study.
